# The prospect of suicide biomarkers: from neurobiology to precision prevention

**DOI:** 10.3389/fpsyg.2026.1757838

**Published:** 2026-03-05

**Authors:** Na Ren, Siqian Zheng, Yixin Zhou, Xiaoling Zhang, Mini Han Wang, Ying Bian

**Affiliations:** 1 State Key Laboratory of Quality Research in Chinese Medicine, Institute of Chinese Medical Sciences, University of Macau, Taipa, Macau SAR, China; 2 Zhuhai People's Hospital (The Affiliated Hospital of Beijing Institute of Technology, Zhuhai Clinical Medical College of Jinan University), Zhuhai, China; 3 Guangdong Provincial Key Laboratory of Tumor Interventional Diagnosis and Treatment, Zhuhai People's Hospital (The Affiliated Hospital of Beijing Institute of Technology, Zhuhai Clinical Medical College of Jinan University), Zhuhai, China; 4 Faculty of Medicine, Chinese University of Hong Kong, Hong Kong SAR, China

**Keywords:** genetic susceptibility, intervention, neurobiology, neuroimaging, prevention, suicide

## Abstract

Suicide remains a global public health crisis, accounting for over 720,000 deaths annually. Despite progress in identifying risk factors and developing theoretical frameworks, suicide continues to be a complex biopsychosocial phenomenon that eludes comprehensive understanding. This review synthesizes current evidence on the neurobiological underpinnings of suicidal behavior, encompassing genetic and epigenetic vulnerabilities, systemic physiological dysregulations, and structural and functional brain alterations. Furthermore, it critically evaluates recent advances in biologically-targeted interventions and assesses the translational potential of biomarker research for precision psychiatry. While these developments provide promising avenues for objective risk stratification and targeted treatments, significant challenges persist in bridging the gap between scientific discovery and clinical implementation. By integrating evidence across these disciplines, this review aims to provide clinicians and researchers with a comprehensive reference to inform clinical practice and guide future research directions.

## 1 Introduction

Suicide remains a critical global public health challenge, with over 720,000 deaths in 2021 (World Health Organization, 2025; Davis Weaver et al., 2025). Among individuals aged 15 to 29, suicide is the third leading cause of death globally, ranking first in several regions (Davis Weaver et al., 2025). Despite decades of research, the persistent burden of suicide underscores the limitations of current prevention paradigms. These approaches primarily rely on self-reported ideation and clinical interviews, which are susceptible to underreporting, lack objectivity, and often fail to account for potential inter-individual biological variability (Turecki and Brent, 2016; Lutz et al., 2017).

**Figure 1:**
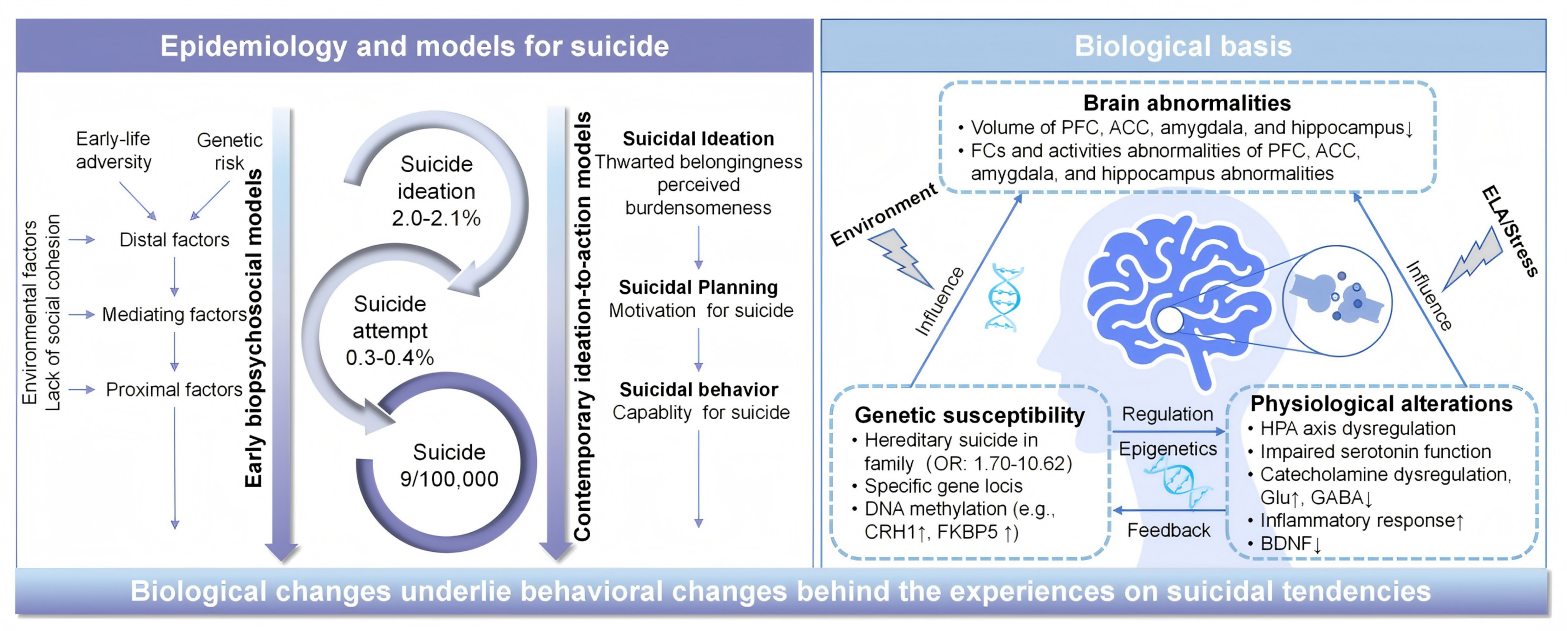
Modeling suicide risk and biological basis of suicide. Estimated global prevalence rates are indicated in each circle. Several models have been proposed to describe the factors that lead individuals to transition from non-suicidal self-injury to other forms of suicidal behavior, including death. These models agree that biological changes underlie behavioral changes behind the experiences on suicidal tendencies. ACC, anterior cingulate cortex; BDNF, brain-derived neurotrophic factor; CRH1, corticotrophin releasing hormone receptor 1; DNA, deoxyribonucleic acid; ELA, early life adversity; FC, functional connectivity; FKBP5, FK506-binding protein 5; GABA, gamma-aminobutyric acid; Glu, glutamate; HPA, hypothalamic-pituitary-adrenal; OR, odds ratio; PFC, prefrontal cortex.

In the last decade, the complexity of suicidal behavior has been extensively explored in theoretical models (Figure 1). Early psychosocial models emphasized the role of distal factors (e.g., psychache, emotional dysregulation, and diathesis-stress) during periods of elevated suicide risk (Lutz et al., 2017). Subsequently, contemporary ideation-to-action frameworks, such as three-step theory (Klonsky et al., 2018), interpersonal-psychological theory (Robison et al., 2024), integrated motivational-volitional model (Dhingra et al., 2016), and fluid vulnerability theory (Bryan et al., 2020), consider suicide as a temporally dynamic process wherein biologically rooted vulnerabilities interact with proximal stressors to drive the transition from thought to action. These models indicated that the trajectory toward suicide is mediated by measurable biological changes underlying behavioral manifestations. Large-scale genetic studies have begun to delineate a polygenic architecture, whereas neuroscience has identified reproducible alterations in brain circuits governing emotional regulation and behavioral inhibition (Lutz et al., 2017). These findings hold promise for heralding a shift from a subjective symptom-based management to an objective precision prevention framework.

However, translating these remarkable scientific discoveries into clinically applicable tools poses significant challenges. The methodological rigor, reproducibility across different populations, and ultimate clinical applicability in guiding treatment decisions all require rigorous evaluation. Therefore, this review, based on a systematic literature search up to 2025 with a focus on large-scale human studies and randomized controlled trials, aims to clarify how a profound understanding of neurobiology can drive suicide prevention toward more objective, personalized, and effective clinical practices, thereby providing a new scientific framework for reducing the social impact of suicidal behaviors.

## 2 Neurobiological biomarkers: from genetic risk to brain

The pursuit of precision suicide prevention is predicated on a multilevel understanding of neurobiological risk. This section synthesizes the current evidence across three domains: (1) genetic and epigenetic vulnerabilities, (2) systemic physiological dysregulations in stress response, neurotransmission, and inflammation, and (3) structural and functional abnormalities in critical brain regions. By evaluating these studies, we aim to delineate a promising path for the biomarker of suicide.

### 2.1 Genetic and epigenetic vulnerabilities

The heritable nature of suicidal behavior is well-established. Convergent evidence from familial, twin, and adoption studies demonstrates a significant genetic component, with heritability estimates ranging from 30% to 50% (Pedersen and Fiske, 2010). Research further indicates that the risk of suicide among relatives of affected individuals remains elevated even after controlling for co-occurring psychiatric diagnoses (Turecki and Brent, 2016), with odds ratios ranging from 1.70 to 10.62. Notably, this transmission appears to be mediated in part by the heritability of intermediate neurocognitive phenotypes, rather than simply imitating behavior (Sokolowski and Wasserman, 2020). Jones et al. found that youth with a family history of a fatal or non-fatal suicide attempt exhibit differences in executive function, attention, and language reasoning compared to youth without a family history (Jones et al., 2021). These findings suggest that genetic risk for suicide is expressed through alterations in cognitive and stress response systems to a degree, predisposing individuals to maladaptive coping under distress.

Recent large-scale genome-wide association studies (GWAS) have identified promising and replicable genetic loci associated with suicide attempts across diverse populations (Kimbrel et al., 2022). Meta-analyses involving over 400 thousand individuals have identified multiple risk loci, with consistent signals implicating genes involved in dopamine signaling and neuronal development (Kimbrel et al., 2022). Docherty et al further conducted the largest GWAS meta-analysis to date (*N* = 958,896; Docherty et al., 2023). This multi-ancestry study identified 12 significant loci contributing to suicide risk, and established significant shared genetic covariation with clinical phenotypes (e.g., attention deficit hyperactivity disorder, smoking, and posttraumatic stress disorder). These findings provide a foundational framework for calculating polygenic risk score (PRS) and understanding the shared genetic architecture with related psychiatric traits.

In addition to specific genes, epigenetic mechanisms also play an important role in suicidal behavior. Epigenetic changes, such as deoxyribonucleic acid methylation, can affect gene expression and function, thereby increasing the risk of suicidal behavior. For example, genetic polymorphisms that interact with childhood abuse and/or neglect, such as corticotrophin releasing hormone receptor 1, FK506-binding protein 5 (FKBP5), etc., may constitute specific mediators between childhood abuse and suicidal tendencies (Lippard and Nemeroff, 2019; González-Castro et al., 2023). The degree of methylation of these genes was positively associated with severe suicidal ideation, suggesting that epigenetic changes may be an important risk factor for suicidal behavior. Emerging evidence highlights the critical role of epigenetic modifications in suicidal behavior through their regulation of stress response and neuroplasticity pathways (Navarro et al., 2023). Notably, studies have shown that abnormal spindle and kinetochore associated complex subunit 2 (SKA2) expression can impair the negative feedback regulation of hypothalamic-pituitary-adrenal (HPA) axis and increase stress sensitivity (Kouter et al., 2023; González-Castro et al., 2024). However, this association seems to show potential ethnic variations, as increased SKA2 expression was found in the Mexican population (Valenzuela-García et al., 2024). Similarly, increased methylation of the neuroplasticity-related gene (e.g., brain-derived neurotrophic factor [BDNF]) is associated with suicidal ideation, attempt history, and poor treatment response, suggesting a molecular substrate for cognitive inflexibility (Navarro et al., 2023). These converging findings suggest that: (1) epigenetic dysregulation of stress response systems (e.g., via SKA2) may heighten vulnerability to suicidal behavior under stress; and (2) impaired neuroplasticity (e.g., via BDNF methylation) may represent a molecular substrate for the cognitive inflexibility characteristic of suicidal states. However, their reproducibility varies among different populations. Without independent validation, the applicability of these biomarkers still needs further exploration.

### 2.2 Systemic physiological dysregulations

The genetic and epigenetic vulnerabilities outlined in the previous section are realized through their impact on downstream physiological systems. These systems do not operate in isolation but form an integrated network whose dysfunction creates a biological context of heightened suicide risk. This section synthesizes evidence on three core pillars of systemic dysregulation: stress response system, monoaminergic and amino acid neurotransmission, and immune-inflammatory signaling, all of which converge to shape suicide risk.

#### 2.2.1 Stress system dysregulation: the HPA axis

The HPA axis, as the primary neuroendocrine stress response system, plays a pivotal role in the pathophysiology of suicidal behavior. Extensive research has demonstrated significant HPA axis dysregulation in individuals at risk for suicide, particularly those with histories of early life adversity (Berardelli et al., 2020). A pattern emerges wherein suicide attempters exhibit paradoxically elevated cortisol concentrations (Chatzittofis et al., 2013). This phenomenon may be related to the dysregulation of the negative feedback mechanism of the HPA axis, potentially mediated by glucocorticoid receptor resistance stemming from chronic HPA axis hyperactivity in traumatized individuals (O'Connor et al., 2018; Underwood et al., 2023). Dexamethasone suppression test provides further evidence of this dysregulation, showing blunted cortisol suppression in suicide attempters indicative of HPA axis feedback impairment (Spaan et al., 2025). Molecular evidence corroborates this, showing altered expression of glucocorticoid receptor and its regulator FKBP5 in the brains of suicide completers (González-Castro et al., 2023). Crucially, this vulnerability is often programmed in early life; polymorphisms in the FKBP5 gene interact with childhood trauma to substantially amplify suicide risk, highlighting a key gene-environment underlying HPA axis dysfunction (Lippard and Nemeroff, 2019; Roy et al., 2017). A key unresolved question is whether HPA axis dysregulation represents a stable, trait-like vulnerability factor or a transient, state-dependent marker of acute crisis. Longitudinal studies are needed to determine if these abnormalities normalize with symptom remission or persist as enduring risk indicators.

#### 2.2.2 Neurotransmitter system dysfunction

Suicide behavior is underpinned by complex dysregulations across multiple neurotransmitter systems and neuroendocrine pathways. Serotonin (5-hydroxytryptamine, 5-HT) system impairment is one of the most replicated findings in suicide research. Its dysfunction is linked to the core behavioral phenotypes of suicide, including impulsivity, aggression, and negative emotional bias (Lutz et al., 2017; Navarro et al., 2023; Sudol and Mann, 2017; Bengoechea-Fortes and Martínez-Martos, 2024). Evidence indicates higher brainstem 5-HT_1A_ receptor, which is associated with reduced serotonin neuron firing and release, potentially leading to impaired regulation of the limbic system (Sullivan et al., 2015). Furthermore, a key gene-environment interaction is observed, wherein the low-expression variant of the serotonin transporter polymorphism (5-HTTLPR) interacts significantly with childhood trauma (Sivaramakrishnan et al., 2023), aligning with the stress-quality model. From a metabolic perspective, decreased cerebrospinal fluid levels of the serotonin metabolite 5-hydroxyindoleacetic acid (5-HIAA) have been widely verified in suicide victims (Wisłowska-Stanek et al., 2021), especially pronounced in females. This suggest that metabolic disorders of 5-HT may increase the risk of suicide by affecting impulse control and emotion regulation (Underwood et al., 2018).

Additionally, dysregulation of the catecholamine system, including norepinephrine and dopamine, has been implicated in suicidal behavior due to its critical role in stress response and reward processing (Sudol and Mann, 2017). Postmortem and clinical studies consistently report reduced cerebrospinal fluid levels of the norepinephrine metabolite 3-methoxy-4-hydroxyphenylglycol and of the dopamine metabolite homovanillic acid in suicide attempters, indicating impaired catecholaminergic neurotransmission (Okada and Fukuyama, 2020). An imbalance between the major excitatory (glutamate, Glu) and inhibitory (gamma-aminobutyric acid, GABA) neurotransmitters is increasingly recognized. Suicidal behavior is associated with a shift toward excitotoxicity, evidenced by increased glutamatergic receptor expression in the prefrontal cortex (PFC) and anterior cingulate cortex (ACC; Zhao et al., 2018). Concurrently, GABAergic deficits may weaken inhibitory control over emotional and behavioral impulses (Choudary et al., 2005). This disrupted Glu-GABA equilibrium likely underlies the aberrant neural excitability and emotional dysregulation observed in suicidal individuals.

#### 2.2.3 Inflammatory response

Growing evidence underscores the pivotal role of inflammatory dysregulation in the pathophysiology of suicidal behavior (Bengoechea-Fortes and Martínez-Martos, 2024; Underwood et al., 2018). Elevated levels of pro-inflammatory cytokines, including interleukin-6 (IL-6), tumor necrosis factor-alpha (TNF-α), and interleukin-1 beta, are consistently observed in both peripheral blood and cerebrospinal fluid of suicide attempters, indicating systemic and central nervous system inflammation (Shinko et al., 2020). Meta-analytic data confirm a dose-dependent relationship: for instance, each 1 mg/L increase in C-reactive protein (CRP) is associated with a 127% higher likelihood of a suicide attempt (Black and Miller, 2015). Inflammatory cytokines may contribute to suicidal behavior through multiple neurobiological mechanisms. On the one hand, pro-inflammatory signaling can shift tryptophan metabolism away from serotonin synthesis toward the kynurenine pathway (KP); on the other hand, inflammatory processes may impair endocannabinoid signaling, which plays a critical role in stress adaptation and emotion regulation (Miller and Raison, 2016; Gunduz-Cinar, 2021). These metabolic and signaling alterations lead to the accumulation of neuroactive metabolites, including the N-methyl-D-aspartate receptor (NMDAR) agonist quinolinic acid. This excessive production promotes excitotoxicity and disrupts the balance between excitatory and inhibitory neurotransmission (e.g., Glu/GABA; Brown et al., 2024). Moreover, prolonged exposure to pro-inflammatory cytokines reduces neurotrophic support, such as BDNF, and impairs synaptic plasticity in brain regions involved in emotion regulation, including the PFC and hippocampus (Calabrese et al., 2014). These alterations ultimately weaken cognitive control over negative affect and suicidal ideation. This impairment is commonly observed in individuals with heightened suicide risk. Although inflammation is correlated with suicide, it remains unclear whether it is a causal driver, a consequence of psychological stress, or an epiphenomenon. Interventions targeting inflammation are needed to establish a causal link and determine if anti-inflammatory strategies can mitigate suicide risk.

### 2.3 Brain structure and function abnormalities

The genetic vulnerabilities and systemic physiological dysregulations detailed in previous sections converge to alter the structure and function of critical brain circuits. A growing body of neuroimaging evidence reveals that suicidal behavior is characterized by distinct structural and functional abnormalities within neural circuits governing emotional regulation, cognitive control, and decision-making processes (Barredo et al., 2021). This section examines how disruptions in key prefrontal and limbic regions and their interconnections create a neural context that facilitates the transition from suicidal ideation to action.

#### 2.3.1 PFC: cognitive control and behavioral inhibition

The PFC, essential for executive control and behavioral regulation, exhibits region-specific impairments in suicide attempters (Sudol and Mann, 2017). Neuroimaging studies reveal region-specific alterations in both ventral (vPFC) and dorsal (dPFC) PFC that contribute differentially to suicide risk pathways (Schmaal et al., 2020). The vPFC, encompassing the orbitofrontal cortex and inferior frontal gyrus, is crucial for impulse control and emotional valuation, which shows structural deficits like gray matter reduction (Fan et al., 2019; Zhao et al., 2021). Functional neuroimaging studies demonstrate vPFC hyperactivation during negative emotional stimuli processing (e.g., angry faces), reflecting impaired emotional regulation that may predispose to impulsive-aggressive behaviors (Huang et al., 2025). These structural and functional abnormalities jointly constitute the emotional dysregulation (e.g., enhanced negative emotions/weakened positive emotions), negative cognition (e.g., rumination/self-blame), and motivational deficits (e.g., helplessness/anhedonia) triad of suicide risk, which is thought to contribute to key clinical risk symptoms for suicide (Schmaal et al., 2020). As the hub for cognitive flexibility and complex decision-making, the dPFC shows hypoactivation during cognitive tasks in suicidal individuals (Barredo et al., 2021). This deficit undermines the ability to generate alternative solutions to crises and maintain cognitive control, lowering the threshold for impulsive suicidal acts. During the development of suicidal behaviors, this dual dysfunction, a hyperactive vPFC coupled with a hypoactive dPFC, creates a perilous imbalance where negative urges are poorly controlled, and adaptive cognitive strategies are inaccessible, thereby promoting the transformation of suicidal ideation into behavior.

#### 2.3.2 ACC: conflict monitoring and emotional evaluation

The ACC, which monitors internal conflict and regulates emotional responses, is consistently implicated in suicide (Schmaal et al., 2020). Structural neuroimaging studies show consistent gray matter volume reductions in suicide attempters, particularly in mood disorder populations (Bani-Fatemi et al., 2018). These structural deficits correlate with the severity and mortality rate of suicidal behavior. At the functional brain, ACC shows characteristic abnormal activation patterns: ventral ACC (vACC) shows excessive activation during emotional processing, whereas dorsal ACC (dACC) shows insufficient activation in cognitive control tasks (e.g., Stroop, Go/No-Go; Bani-Fatemi et al., 2018). This functional imbalance reflects the emotional regulation disorder and executive function defect behind suicidal behavior. In terms of emotional information processing, the response of ACC to negative stimuli (e.g., angry faces) is enhanced, whereas the response to positive stimuli (e.g., reward expectations) is weakened. This abnormal pattern of emotional processing may lead to the amplification of negative emotions and the weakening of positive experiences, thereby promoting the formation of suicidal ideation. Meanwhile, the impaired function of dACC in conflict monitoring and behavior suppression will reduce the threshold of behavior control and facilitate impulsive suicidal acts.

#### 2.3.3 Amygdala and hippocampus: salience processing and contextual memory

The amygdala and hippocampus, central to threat detection and contextual memory, show significant alterations. Studies have shown that the volume of the amygdala and the hippocampus is significantly reduced in people who have attempted suicide (Vieira et al., 2023). These structural changes may be associated with dysregulation of emotions and memory processing, leading to increased impulsivity and aggression in suicidal behavior. Functional magnetic resonance imaging studies have shown that the response to negative stimuli is weakened (Vai et al., 2023). This may not indicate a lack of emotion but rather a blunted salience processing or emotional exhaustion, which is a feature of severe suicide risk. Huang et al. (2024) further found that individuals with greater alexithymic traits exhibited increased functional connectivity between the amygdala and the occipital face area during facial stimulus processing, yet decreased connectivity during emotional processing. These findings suggest that the neurobiological underpinnings of suicide risk may involve not only hypoactivation within limbic structures but also disrupted integration between the amygdala and cortical regions during affective processing. These connectivity alterations may contribute to emotional and memory processing dysregulation, thereby increasing impulsivity and aggression in suicidal behavior.

### 2.4 Toward an integrative neurobiological model of suicide

The evidence converges on a multilevel model of suicide vulnerability (Figure 1). Genetic predisposition and early-life adversity conspire, via epigenetic mechanisms, to calibrate the stress response system (HPA axis dysregulation) and alter neurodevelopment. These effects manifest in adulthood as systemic physiological imbalances, including serotonin deficiency, inflammation, and disrupted Glu/GABA signaling, which in turn disrupts the structure and function of key brain circuits. The core pathology involves dysfunctional prefrontal-limbic connectivity: a hyperactive, emotionally salient limbic system (e.g., amygdala) coupled with a hypoactive cognitive control system (e.g., dPFC, dACC) and a dysregulated evaluative system (e.g., vPFC, vACC). This neural circuit failure underlies the clinical phenotype of cognitive inflexibility, impaired impulse control, and unremitting psychological pain that defines acute suicide risk.

## 3 Biomarker-guided interventions for suicide prevention

**Figure 2:**
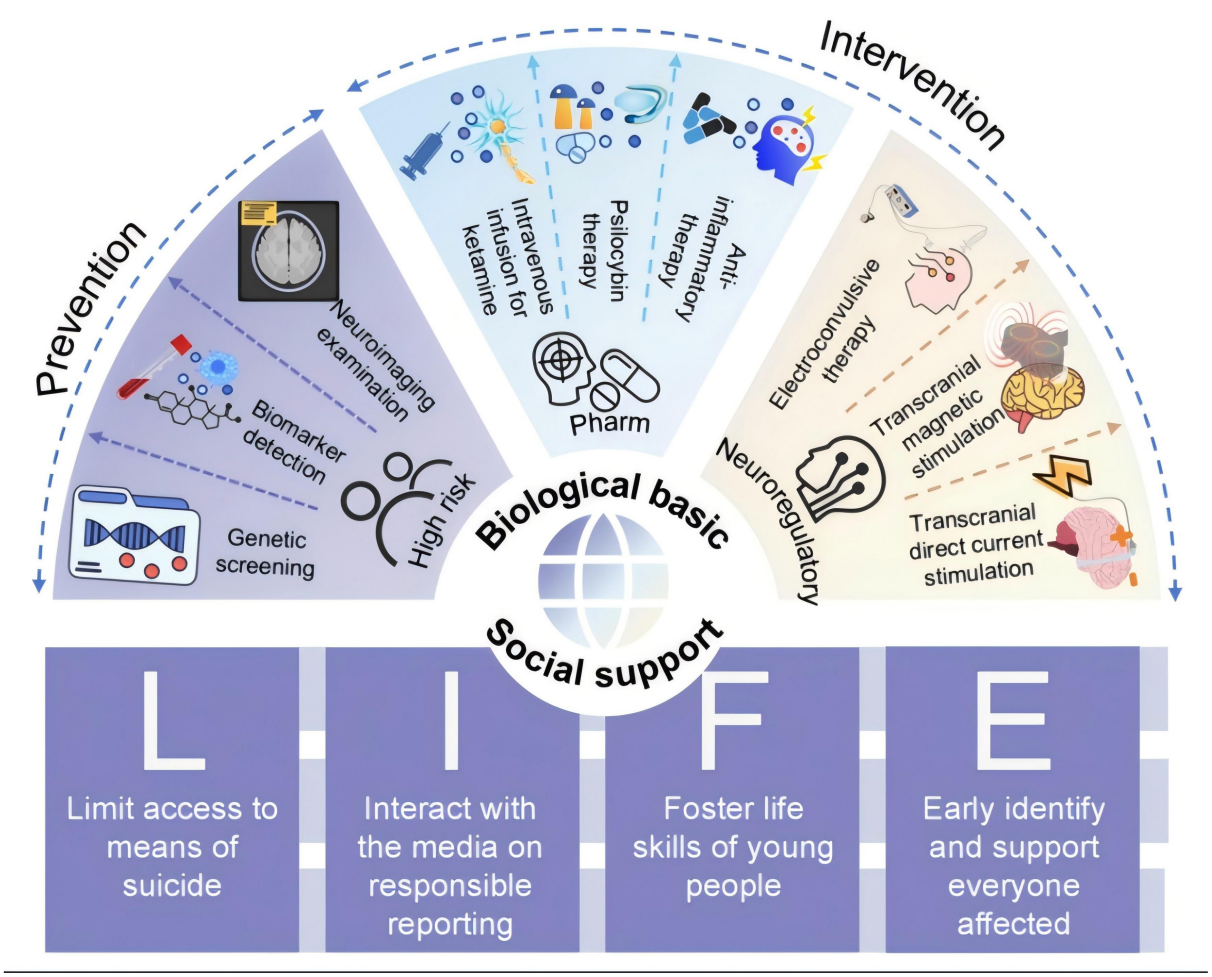
Suicide prevention and intervention strategy framework. This schematic outlines complementary strategies grounded in social supports and biological basic. The social support component incorporates the evidence-based “LIFE” intervention measures recommended by the World Health Organization for preventing suicide and self-harm. Advances in understanding the biological basis of suicide have enabled a translational pipeline that spans from biomarker prevention to targeted interventions. Specific biomarkers and targeted interventions (e.g., pharmacotherapies and neuromodulation) referenced in this framework are elaborated in subsequent sections.

Advances in understanding the neurobiology of suicide are driving a paradigm shift from reliance on subjective assessment toward biomarker-informed strategies. As illustrated in Figure 2, this approach advances along two complementary areas: the implementation of population-level, evidence-based “LIFE” strategies recommended by the World Health Organization (e.g., limiting means, media guidelines), and the development of targeted interventions grounded in the specific genetic, molecular, and neural circuit dysregulations associated with suicide risk. Corresponding to this framework, the following sections first review advances and challenges in applying biomarkers for risk stratification, and then systematically examine emerging mechanism-based interventions.

### 3.1 Risk assessment for suicide prevention

Current clinical risk assessment mainly relies on self-reported scales (e.g., Beck Scale for Suicide Ideation, Columbia-Suicide Severity Rating Scale), which is subject to recall bias and intentional concealment. Current assessment is undergoing a paradigm shift from single self-report to multimodal integration (Table 1).

**Table 1:** Clinical scales and emerging methods for suicide risk assessment.

	**Assessment Method**	**Assessment dimension**	**Application scenarios**	**Advantages and limitations**
Current clinical practice	Beck scale for suicide ideation	Assesses severity of current suicidal ideation, including frequency, intensity, duration, and control over suicidal behavior.	In-depth evaluation of individuals with identified suicide risk in clinical settings.	Advantages: high standardization, good reliability and validity. Limitations: relies on self-report, susceptible to recall bias and deliberate concealment.
	Columbia-suicide severity rating scale	Systematically evaluates suicidal ideation, suicidal behavior, and their severity.	Multiple scenarios including emergency departments, psychiatric clinics, and clinical trials; available in screening, recent, and lifetime versions.	Advantages: structured assessment, distinguishes between ideation and behavior. Limitations: still requires patient self-report, potential information bias.
	Suicidal behaviors questionnaire-revised	Assesses lifetime suicidal ideation/attempts, recent suicide frequency, communication of suicide threats, and future suicide probability.	Rapid screening in large populations.	Advantages: concise and efficient (only 4 items). Limitations: limited depth of assessment.
	Suicide items in depression scales	Routine screening in depressed patients	Routine screening in depressed patients.	Advantages: easy to administer, integrated into routine assessment. Limitations: limited scope of assessment, lacks detail.
Emerging approaches	Genetic biomarkers	Polygenic risk estimation based on GWAS summary statistics	Genetic risk stratification in high-risk populations.	Advantages: objective quantification of genetic risk. Limitations: limited individual prediction accuracy, requires combination with environmental and clinical factors.
	Molecular biomarkers	Inflammatory markers (CRP, IL-6), HPA axis indicators (cortisol), metabolites, etc.	Auxiliary diagnosis and risk stratification.	Advantages: provides window into pathophysiology. Limitations: limited predictive power of single biomarkers; state-dependent variability limits longitudinal prediction.
	Neuroimaging biomarkers	Structural and functional abnormalities in prefrontal-limbic circuits	Brain mechanism research at systems level.	Advantages: provides objective evidence of neural system abnormalities. Limitations: high cost; technical barriers.
	Multimodal integrated models	Combines behavioral, genetic, molecular, and neuroimaging data using machine learning approaches	Precise risk assessment and prediction.	Advantages: significantly improves prediction accuracy Limitations: complex data integration; limited interpretability and lack of prospective clinical validation.

At the genetic level, PRS, derived from integrating GWAS data, show promise for improving population-level risk stratification. For example, when applied in participants recruited from the psychiatric emergency department, the addition of PRS improved identification performance from an area under the curve (AUC) of 0.84 to 0.86 (Lee et al., 2024), highlighting its incremental value within multivariate models. Furthermore, specific genetic variants, such as those in FKBP5 and 5-HTTLPR, have demonstrated utility in predicting short-term suicide risk, particularly among individuals with a history of childhood trauma (Lippard and Nemeroff, 2019; Sivaramakrishnan et al., 2023). These underscore the potential value of genetic profiling in subgroups with specific environmental exposures, although its standalone clinical applicability remains limited.

At the molecular level, peripheral biomarkers, including inflammatory markers, cortisol, and metabolic profiles, provide a dynamic window into pathophysiology and show promise in differentiating suicidal states (Navarro et al., 2023). For instance, changes in cortisol, tryptophan, and endogenous cannabinoid levels have demonstrated unique value in differentiating different stages such as suicidal ideation, attempted suicide, and suicidal death (Johnston et al., 2022). However, the diagnostic performance of a single biomarker remains relatively limited. The AUC values reported for metabolomics and proteomics features distinguishing suicide attempters from healthy controls were 0.601 and 0.702, respectively (Zhang et al., 2024), a range generally considered to provide limited clinical utility. By contrast, a behavior-based classification model reported an AUC of 0.909, highlighting the substantially greater discriminative power of behavioral information. This discrepancy further underscores the necessity of developing multimodal integrated models, in which biological biomarkers are incorporated alongside behavioral and clinical data to achieve clinically meaningful predictive performance.

In the field of neuroimaging, structural and functional abnormalities within prefrontal-limbic circuits provide a systems-level biomarker (Schmaal et al., 2020; Vieira et al., 2023). Advances in artificial intelligence based image analysis techniques have substantially improved the detection of complex and high-dimensional neuroimaging patterns, making the clinical application of these biomarkers possible. However, reported classification performance varies markedly across studies, with AUC values ranging from 0.52 to 0.989 (Parsaei et al., 2023). This substantial variability is primarily attributable to methodological heterogeneity, including differences in sample size, imaging modality, feature selection strategies, machine learning algorithms, validation procedures (e.g., internal cross-validation or independent test sets), and characteristics of the study populations. For example, near-perfect AUC values (e.g., >0.95) reported in studies with small samples are highly likely to reflect overfitting, in which models capture dataset-specific features rather than generalizable biological signals. Although such findings may demonstrate technical feasibility under constrained experimental conditions, they should not be interpreted as evidence of clinical readiness. Additionally, a recent study based on the large Adolescent Brain Cognitive Development cohort constructed a prediction model with neuroimaging features and reported an AUC of 0.52, a value close to random classification (van Velzen et al., 2022). This limited performance may be influenced by several factors, including the relatively young age of the participants, the use of a population-based sample, and inherent methodological constraints (Bissett et al., 2021). Collectively, these findings underscore the critical importance of sample characteristics and rigorous validation in interpreting reported model performance. They further highlight that the field remains in a developmental stage. Future studies should prioritize larger sample sizes, multicenter collaborations, standardized data acquisition protocols, and rigorous validation frameworks to distinguish stable predictive signals from methodological bias, thereby clarifying the potential clinical applicability.

The current field of suicide risk assessment is undergoing a significant paradigm shift. Although traditional clinical scales have been widely validated and play an important role in practice, their assessment effectiveness is limited by the subjectivity of patient reports. At the same time, emerging biomarkers provide objective supplementary evidence from genetic, molecular, and systemic levels, showing promising potential. However, the independent predictive ability of most biomarkers is still limited, insufficient to support precise individual-level diagnosis. Therefore, the development of comprehensive multimodal assessment systems that integrate clinical scales with multidimensional biological markers has become increasingly important. One commonly adopted strategy is feature-level fusion, in which features derived from different modalities, such as PRS, neuroimaging-derived measures, and behavioral or clinical variables, are first standardized and then concatenated into a unified feature space for joint modeling using regularized regression or machine learning algorithms (Hao et al., 2023; Huang et al., 2026). This approach enables the identification of potential cross-modal interactions but requires careful dimensionality reduction and regularization to mitigate the risk of overfitting associated with high-dimensional data. An alternative strategy is decision-level fusion, whereby separate predictive models are constructed for each modality, and their outputs (e.g., estimated risk probabilities) are subsequently integrated using predefined rules (e.g., weighted averaging, voting schemes, or meta-learners) to generate a final composite risk estimate (Pigoni et al., 2024). The advantage of this approach lies in its flexibility, allowing each modality to be analyzed using methods best suited to its data characteristics. Although multimodal assessment systems remain largely at the research and validation stage, they provide a structured pathway toward more comprehensive, robust, and reliable suicide risk assessment.

### 3.2 Targeted biological interventions

#### 3.2.1 Progress in pharmacotherapy

In contrast to conventional antidepressants, novel pharmacotherapies aim to directly modulate the neurobiological pathways implicated in suicide, particularly in high-risk psychiatric patient populations. As an NMDAR antagonist, ketamine has demonstrated remarkable rapid-onset anti-suicidal effects, primarily studied in patients with treatment-resistant depression (TRD; Mallick and McCullumsmith, 2016). Clinical studies show that a single intravenous infusion (0.5 mg/kg over 40 min) can produce significant reductions in suicidal ideation within 24 h (Xiong et al., 2021; Wilkinson et al., 2017). These rapid effects are thought to occur through restoration of prefrontal synaptic plasticity, increased BDNF levels, and normalization of reward network connectivity (Krystal et al., 2024). Current clinical practices suggested 6 infusions over 2–3 weeks for sustained response, which makes 69% of participants experience complete alleviation of suicidal ideation following repeated infusions (Phillips et al., 2020; McIntyre et al., 2021). Although effective for acute stabilization, ketamine requires cardiac monitoring due to potential QTc prolongation.

Psilocybin therapy represents another innovative approach, leveraging 5-HT_2A_ receptor-mediated cortical plasticity and regulation of brain functional activities (Ling et al., 2022). Its anti-suicidal effects have been investigated in patients with major depressive disorder (MDD) and bipolar II disorder, a population exhibiting a high prevalence of suicidal ideation (Aaronson et al., 2024; Daws et al., 2022). Psilocybin therapy (25 mg combined with psychological support) has demonstrated both rapid (24–48 h) and sustained (≥4 week) reductions in suicidal ideation (Daws et al., 2022). The therapeutic effects correlate with increased cortical entropy and lasting changes in amygdala-ventromedial PFC connectivity (Daws et al., 2022). However, the long-term safety still needs more research. The intervention requires careful patient selection and extensive therapeutic support, with particular caution needed for patients with psychotic disorders (Goodwin et al., 2022).

For patients exhibiting evidence of neuroinflammation (CRP >3 mg/L or IL-6 >2 pg/mL), anti-inflammatory approaches have shown promise. TNF-α inhibitors like etanercept can reduce suicidal ideation scores compared to placebo, potentially mediated by reduced neuroinflammation and quinolinic acid production (Kappelmann et al., 2018). Novel therapies targeting the KP present a promising mechanism-based approach for suicide prevention, particularly for suicide risk associated with TRD. KP metabolism is dysregulated in TRD and suicidal states, characterized by a shift toward the neurotoxic branch marked by increased production of the NMDAR agonist quinolinic acid, and away from the neuroprotective branch marked by reduced production of the NMDAR antagonist kynurenic acid (Serafini et al., 2017). This imbalance can lead to glutamatergic excitotoxicity and impaired neuronal integrity, which are key pathological processes closely linked to suicidal behavior. Minocycline, an antibiotic with anti-inflammatory and neuroprotective properties, shows an effect on KP modulation. Minocycline may rebalance this pathway by suppressing the neuroinflammation-driven induction of the rate-limiting enzyme indoleamine 2,3-dioxygenase. Preliminary clinical observations indicate that symptom improvement, particularly the reduction in suicidal ideation following minocycline treatment, correlates with favorable alterations in KP metabolite ratios (Nettis et al., 2023). Although these novel approaches require further validation through large-scale clinical trials, they represent a significant shift toward a “pathophysiology-specific” precision treatment paradigm.

#### 3.2.2 Progress in neuromodulation

Neuromodulation techniques offer a direct approach to modulating dysfunctional brain circuits (Chen et al., 2021). Electroconvulsive therapy remains the most effective rapid-acting intervention for acute suicidal crises in patients with MDD and TRD, with over 75% of high-risk patients showing remission of suicidal ideation after a course of treatment (Naismith et al., 2025; Serafini et al., 2020). As a non-invasive alternative, high-frequency (10 Hz) repetitive transcranial magnetic stimulation applied to the dorsolateral PFC has demonstrated a 40%-50% reduction in suicidal ideation in TRD patients (Serafini et al., 2020). This effect is thought to be a result of the normalization of connectivity between the PFC and the amygdala, as well as the enhancement of top-down cognitive control. A significant barrier is the limited accessibility and availability of these neuromodulation technologies outside specialized centers. Furthermore, the precise neural mechanisms underlying their therapeutic effects are still being elucidated, indicating a need for more refined and personalized stimulation protocols.

#### 3.2.3 Biomarker-guided interventions within the broader prevention landscape

Although this review focuses on the potential of biomarker-guided interventions in suicide risk management, it must be acknowledged that the strongest and most extensive current evidence for reducing suicidal behavior comes from psychosocial and behavioral interventions. These measures form the cornerstone of suicide prevention practices, mainly including: (1) safety planning intervention, which involves patients in a suicide narrative to identify individual warning signs and to provide a predetermined set of potential coping strategies and social supports; (2) restricting access to lethal means; (3) brief contact intervention, which provides easily accessible and continuous support through one session or via several brief contacts, such as caring contacts that express caring concern to suicidal patients following discharge from inpatient treatment by routinely sending supportive information; (4) collaborative assessment and management of suicidality, a therapeutic framework centered on working directly with suicidal ideation, which guides the assessment, management, and treatment planning of suicide risk by establishing a strong collaborative relationship and co-developing personalized safety strategies (Turecki et al., 2019; Zalsman et al., 2016). Practice has shown that these interventions can effectively reduce the incidence of suicide attempts, suicidal ideation, and related behaviors. In contrast, biomarker-guided interventions are currently still in the research and development stage. Their goal is not to replace these established strategies, but to serve as complementary tools for enhancing risk stratification and personalizing treatment selection, thereby mitigating the underlying neurobiological heterogeneity that affects individuals' responsiveness to psychosocial interventions. Future suicide prevention models should adopt a multi-level strategy that combines effective psychosocial and behavioral interventions with emerging tools such as biomarkers, to establish a comprehensive prevention system spanning from public health to individual clinical care.

## 4 Discussion

This review has synthesized promising evidence that suicidal behavior is anchored in a multilevel neurobiological framework, encompassing genetic and epigenetic vulnerabilities, systemic physiological dysregulations, and distinct brain circuit abnormalities. The convergence of findings across these domains confirms that suicide risk is expressed through measurable biological pathways, thereby affirming its biological plausibility. This integrated framework also provides a conceptual roadmap for developing targeted interventions. The critical challenge facing the field has shifted from the confirmation of biological associations to the translation of scientific knowledge into reliable, life-saving clinical tools.

However, achieving this vision is still limited by several key practice gaps. The primary obstacle is the severe lack of clinical utility evidence: although candidate biomarkers are constantly emerging, the field lacks robust, well-designed prospective clinical trials and remains mainly at the discovery and validation stage. Consequently, it remains unclear whether biomarker-based interventions can effectively improve key clinical endpoints (such as suicide attempts or mortality), which makes many current translational claims premature. Secondly, the fragmentation of research hinders the development of comprehensive theoretical models. Suicidal behavior is the result of complex interactions of biological, psychological, and social factors (Zhang et al., 2024); however, currently most studies are still limited to a single dimension, lacking models that can systematically integrate multi-level data such as genes, brain imaging, clinical psychology, and social environment, limiting the accuracy of risk prediction and the completeness of mechanism understanding. Finally, reproducibility and universality pose fundamental bottlenecks for transformation. Many initially promising biomarkers (e.g., SKA2 epigenetic markers or specific brain functional connectivity indicators) show significant differences or contradictory results in different racial, cultural, and socioeconomic backgrounds (Valenzuela-García et al., 2024). Most biomarkers overlap with the general symptoms of mental disorders; and whether these biomarkers reflect state or trait characteristics of suicide risk remains to be determined.

Beyond these scientific and methodological challenges, the clinical implementation of biomarker-based strategies faces practical and ethical hurdles. The cost-effectiveness of widespread biomarker testing, the requirement for specialized expertise in interpretation, and the integration into existing clinical workflows present substantial barriers to adoption. Additionally, the ethical implications of biomarker-based risk stratification demand careful consideration of potential impact of false-positive results.

To address these challenges, the coordinated strategic roadmap in the future should prioritize three critical directions. First, advancing biomarker-guided intervention trials represents the most pressing need. The field must design and implement rigorous prospective trials that test whether biomarker-stratified treatment allocation yields superior outcomes compared to standard care. These studies should specifically target biomarkers with strong preliminary evidence, such as prefrontal-limbic circuit indicators for neuromodulation approaches. Second, developing integrative computational models through large-scale multimodal data integration is essential. Leveraging advanced computational methods (e.g., machine learning and artificial intelligence) to synthesize genetic, molecular, neuroimaging, clinical, and social data will enable the development of more powerful risk prediction tools that reflect the true complexity of suicide risk. Third, establishing ethical implementation frameworks must proceed in parallel with technological advances. This includes creating standardized protocols for biomarker assessment, and developing guidelines for responsible communication of biomarker-based risk information.

In conclusion, the multilevel framework established in this review not only validates the biological plausibility of suicide risk but also provides a conceptual roadmap for developing targeted interventions. Realizing this potential road will require collaboration across disciplines. By addressing the methodological challenges, pursuing the outlined research priorities systematically, and developing ethical implementation frameworks proactively, the substantial scientific foundation would be transformed into clinically useful tools, thereby reducing the global burden of suicidal behavior.
